# Bilateral Olecranon and Left Lesser Trochanter Avulsion Fractures in Renal Osteodystrophy: A Rare Case Report and Comprehensive Review

**DOI:** 10.15388/Amed.2025.32.2.1

**Published:** 2025-12-30

**Authors:** Vijay Ram Kumar Papineni, Rajesh Botchu, Kartikeyan P. Iyengar, Shashank Chapala, Asad Rabbani Shah, Balamurugan Rathinavelu

**Affiliations:** 1 Sheikh Shakhbout Medical City, Abu Dhabi, United Arab Emirates; 2 Royal Orthopaedic Hospital, Birmingham, United Kingdom; 3 Southport and Ormskirk Hospital NHS Trust, Southport, United Kingdom; 4 Asian Institute of Gastroenterology, Hyderabad, India; 5 Sheikh Shakhbout Medical City, Abu Dhabi, United Arab Emirates; 6 Sheikh Shakhbout Medical City, Abu Dhabi, United Arab Emirates

**Keywords:** avulsion fractures, femur, olecranon process, Chronic Kidney Disease-Mineral and Bone Disorder, radiography, ultrasonography, Magnetic Resonance Imaging, avulsiniai lūžiai, šlaunikaulis, olekranono atauga, lėtinė inkstų liga – mineralų ir kaulų sutrikimas, radiografija, ultragarsinis tyrimas, magnetinio rezonanso tomografija

## Abstract

*Chronic Kidney Disease-Mineral and Bone Disorder* (CKD-MBD) disrupts phosphorus and calcium metabolism, leading to skeletal disorders such as demineralization, weakened bones, and an increased fracture risk. These disorders, known as renal osteodystrophy, vary widely and are classified into secondary hyperparathyroidism, adynamic bone, and osteomalacia based on bone turnover. Fracture risk increases with the severity of CKD, particularly in advanced stages (creatinine clearance <15–20 mL/min), but even patients with mild to moderate CKD are at risk of fractures. Fractures of the *Lesser Trochanter* (LT) in adults are rare, and most are caused by underlying malignancies. We present a case of simultaneous avulsion fractures in the left lesser trochanter and bilateral olecranon processes secondary to minor trauma in a young patient with End Stage Renal Disease, which has not been previously reported, and review the literature. This review summarizes the role of imaging (Radiography, US, Computed Tomography, and Magnetic Resonance Imaging) in assessing avulsion fractures in patients with end-stage renal disease. Ultrasound serves as a supportive tool in evaluating the neuromusculoskeletal system; however, in patients with end-stage renal disease, radiography, CT, and MRI remain the primary imaging modalities for assessing avulsion fractures.

## Abstract (it)

Lėtinė inkstų liga (LIL) – mineralų ir kaulų liga (LKN-MBD) sutrikdo organizme fosforo ir kalcio apykaitą, todėl atsiranda skeleto sutrikimų, pavyzdžiui, demineralizacija, silpnėja kaulai ir didėja lūžių rizika. Šie sutrikimai, vadinami inkstų osteodistrofija, yra labai įvairūs ir pagal kaulų apykaitą skirstomi į antrinį hiperparatiroidizmą, adynaminį kaulą ir osteomaliaciją. Lūžių rizika didėja didėjant LIL sunkumui, ypač pažengusiose stadijose (kreatinino klirensas <15–20 ml/min), tačiau lūžių rizika gresia net ir lengvo ar vidutinio sunkumo LIL sergantiems pacientams. Mažojo trochanterio (LT) lūžiai suaugusiesiems pasitaiko retai, o daugumą jų sukelia pagrindinės piktybinės ligos. Pateikiame jaunam pacientui, sergančiam galutinės stadijos inkstų liga, vienu metu įvykusį kairiojo mažojo trochanterio ir abipusių olekranono ataugų avulsinių lūžių atvejį, apie kurį anksčiau nebuvo pranešta, ir apžvelgiame literatūrą. Šioje apžvalgoje apibendrinamas vaizdų (rentgenografijos, kompiuterinės tomografijos ir magnetinio rezonanso tomografijos) vaidmuo vertinant avulsinius lūžius pacientams, sergantiems galutinės stadijos inkstų liga. Ultragarsas yra pagalbinė priemonė vertinant nervų-raumenų-skeleto sistemą, tačiau pacientams, sergantiems galutinės stadijos inkstų liga, radiografija, kompiuterinė tomografija ir magnetinio rezonanso tomografija tebėra pagrindiniai vaizdinimo būdai vertinant avulsinius lūžius.

## Introduction

The olecranon process of the ulna articulates with the trochlear notch of the distal humerus to create a stable ulnohumeral joint which resists forward translational forces and aids in rotational stability. The olecranon is the most prominent part of the elbow joint posteriorly and serves as the anchor point for the triceps muscle. Olecranon fractures usually occur from direct blows from falls or road traffic accidents; however, they have been reported with strong muscular contractions often involving the triceps muscle [1].

The term ‘Atraumatic fracture’ refers to a break in the bone caused by a low-energy force that is incapable of causing a fracture under normal circumstances [2]. This category includes various types of fractures, such as pathological fractures (caused by a weakened trabeculae of bone by disease process), stress fractures (resulting from repeated rhythmic stress on a bone) [3], and fragility fractures (a specific type of insufficiency fracture in osteoporotic bones caused by a minimal traumatic event) [2]. These atraumatic fractures often present with subtle symptoms and may not be visible on imaging, thus making diagnosis challenging due to their occult nature [4]. Lesser trochanter fractures are rare in adults, and are often associated with focal metastatic involvement [5].

## Case report

A 31-year-old male with a known history of *End-Stage Renal Disease* (ESRD) on haemodialysis via an *arteriovenous* (AV) fistula presented to the emergency department following a minor motorcycle accident. He reported falling at a speed of approximately 10 km/hr, landing on his left side, and complained of pain in both elbows and the left hip. The primary survey was unremarkable for any other major thoraco-abdominal or spinal injury.

The *Focused Assessment with Sonography for Trauma* (FAST) scan was negative. The secondary survey revealed pain, swelling, and severe tenderness over the left olecranon and limited movement with a palpable gap in the posterior aspect, suggesting a possible tendon injury. The patient was unable to extend his left elbow against gravity. The right elbow was also painful with mild swelling and bruising, but had a full active range of motion. There was pain and tenderness in the left hip exacerbated by extreme flexion and rotation.

**Figure 1:**
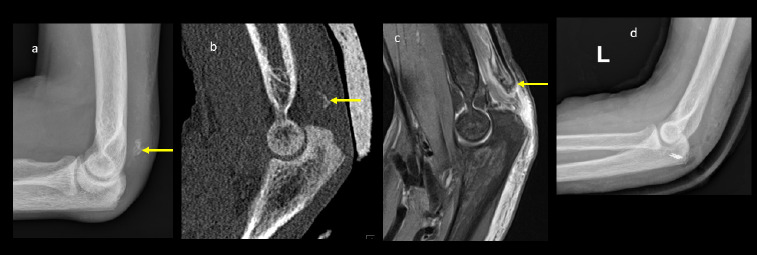
Left elbow a) lateral plain radiograph b) CT sagittal reformats c) Sagittal PD FAT SAT MRI showing avulsion fracture of olecranon (triceps attachment site) with associated posterior soft tissue swelling. The left sided avulsion (yellow arrow) fracture is more displaced compared to the right. Ultrasound scan of the left elbow was not performed due to presence of back slab on the left. d) Post operative plain lateral radiograph of left elbow showing screw fixation of olecranon avulsion fracture.

**Figure 2:**
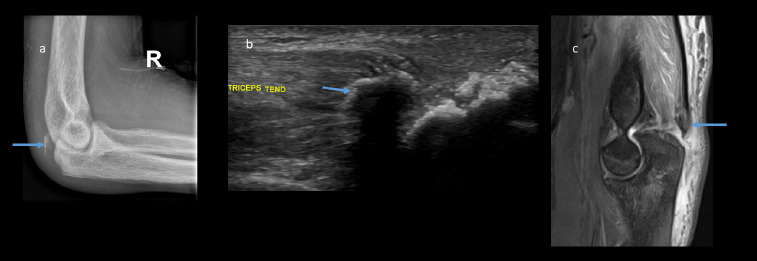
Right elbow a) lateral plain radiograph b) Longitudinal ultrasound c) Sagittal PD FAT SAT MRI showing avulsion fracture of olecranon (blue arrow) from triceps attachment site with associated posterior soft tissue swelling. The right sided avulsion fracture is less displaced compared to the right.

**Figure 3:**
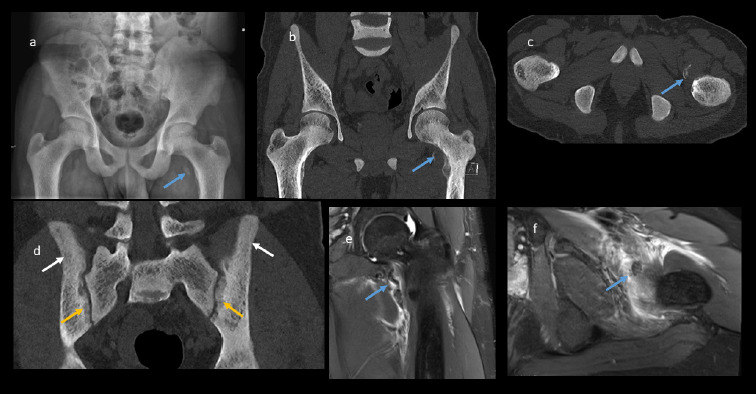
a) plain radiograph of pelvis, b) coronal c) axial CT reformats of pelvis showing avulsion fracture of the left lesser trochanter (blue arrow) and d) coronal reformats of both SI joints showing diffuse bony sclerosis (white arrow) and sub periosteal bone resorption (orange arrow) of both SI joint. e, f) Coronal and axial PD FAT SAT MRI showing avulsion fracture (blue arrow) of left lesser trochanter (psoas attachment site) with associated extensive soft tissue swelling and oedema.

Plain radiographs showed significant bilateral olecranon soft tissue swelling with faint curvilinear ossification proximally suspicious for bilateral avulsion fractures. The ultrasound confirmed loss of tension of the triceps tendon attached to a small bone fragment on the right (Figures 1 and 2). Pelvis radiographs showed an avulsion fracture of the lesser trochanter of the left femur and marked bony sclerosis consistent with renal osteodystrophy. CT of the left elbow indicated a small bony fragment in the distal triceps tendon area with cortical irregularity and significant soft tissue swelling. CT pelvis revealed diffusely increased bone density, subchondral erosions, and resorption at the sacroiliac joints as well as femoral necks consistent with secondary hyperparathyroidism due to end-stage renal disease. MRI of the hip showed generalized skeletal sclerosis along with avulsion of the left iliopsoas tendon with proximal retraction and adjacent soft tissue oedema (Figure 3).

The overall diagnosis included bilateral triceps avulsion fractures with minimal retraction on the right side and significant retraction on the left, a left iliopsoas tendon avulsion with retraction, and florid features of renal osteodystrophy associated with ESRD. The patient underwent surgical repair of the left olecranon avulsion fracture (Figure 1d) while the right olecranon and left lesser trochanter avulsion fractures were managed conservatively.

The patient’s laboratory results reflect significant disturbances associated with end-stage renal disease (ESRD) and secondary hyperparathyroidism. His calcium level was low-normal at 1.94 mmol/L, while phosphate was markedly elevated at 2.3 mmol/L, which is consistent with renal impairment and disrupted mineral metabolism. *Elevated Parathyroid Hormone* (PTH) at 159 pmol/L indicated secondary hyperparathyroidism, which is a common complication in ESRD leading to renal osteodystrophy. Serum alkaline phosphatase was significantly raised at 580 IU/L, suggesting high bone turnover. The patient’s creatinine (Cr) was critically elevated at 1364 micromols/L, with an *Estimated Glomerular Filtration Rate* (eGFR) of 4 mL/min. Albumin (Alb) was low at 22 g/L, indicating poor nutritional status with slightly reduced hemoglobin (Hb) at 110 g/L, reflecting anaemia of chronic disease. The normal *White Blood Cell* (WBC) count and platelets suggested no acute infection or haematological disorder. These findings collectively highlight the severe systemic impact of ESRD on bone and mineral metabolism, contributing to the patient’s increased fracture risk and complications from his injuries.

## Discussion

Renal osteodystrophy, a bone disorder linked to CKD-MBD (Chronic Kidney Disease-Mineral and Bone Disorder), is divided into high-turnover and low-turnover states, with a third category known as mixed uremic osteodystrophy, which includes characteristics of both turnover types and abnormal mineralization [6] (Table 1).

**Table 1:** Features of various types of Renal osteodystrophy

Feature	High Bone Turnover (Osteitis Fibrosa Cystica)	Osteomalacia (Low Bone Turnover)	Adynamic Bone Disease (Low Bone Turnover)	Mixed Uremic Osteodystrophy
**Underlying Cause**	Elevated PTH (secondary hyperparathyroidism)	Heavy metal toxicity (aluminium), vitamin D deficiency, impaired mineralization	PTH suppression, excessive calcium and vitamin D treatment, diabetes	Combination of high and low turnover states
**PTH Levels**	Elevated	May be mildly elevated or normal	Suppressed	Elevated, but not as high as in osteitis fibrosa
**Calcium Levels**	Normal or low-normal	Low due to defective mineralization	Often elevated due to excessive calcium supplementation	Low-normal to elevated
**Phosphate Levels**	Elevated due to reduced renal clearance	Elevated in ESRD or low in cases of vitamin D deficiency	Low or normal, depending on use of phosphate binders	Elevated
**Alkaline Phosphatase (Alk Phos)**	Elevated due to high bone turnover	Elevated due to defective bone mineralization	Normal or low due to very low bone turnover	Elevated but variable depending on the balance of high/low turnover
**Bone Turnover**	Increased bone resorption and formation (high turnover)	Low turnover with defective bone mineralization	Very low turnover with impaired bone remodelling	Mixed – both increased resorption and impaired mineralization
**Bone Markers**	Elevated (high resorption markers)	Elevated due to impaired mineralization	Low due to suppressed bone activity	Variable (reflects both high and low turnover)
**Complications**	Bone pain, deformities, fractures, brown tumours (severe cases)	Soft bones, increased risk of fractures	Increased fracture risk, low bone density	Increased fracture risk, difficult to treat due to dual pathology
**Treatment Approach**	Phosphate binders, vitamin D analogues, calcimimetics, surgery (severe)	Correct vitamin D deficiency, avoid aluminium exposure	Reduce calcium and vitamin D intake, careful management of PTH levels	Balancing treatments for both high and low turnover

High bone turnover results from elevated *Parathyroid Hormone* (PTH) levels and is commonly associated with secondary hyperparathyroidism and osteitis fibrosa cystica. In this state, bone resorption and formation are both increased and in advanced cases, brown tumours may develop due to osteoclastic proliferation along with hemosiderin deposits [6].

Low bone turnover involves conditions such as osteomalacia and adynamic bone disease. Osteomalacia occurs due to heavy metal toxicity, particularly from aluminium, which disrupts proper bone mineralization. Adynamic bone disease, on the other hand, stems from suppressed PTH levels, leading to a reduced bone turnover and inadequate mineralization. Contributing factors include excessive calcium and vitamin D supplementation, peritoneal dialysis, and diabetes [6].

Mixed uremic osteodystrophy, the third form, blends elements of high-turnover bone disease, like osteitis fibrosa, with low-turnover issues seen in osteomalacia. This condition reflects the complicated relationship between an abnormal bone turnover and mineralization in CKD, leading to various skeletal abnormalities. Patients with mixed uremic osteodystrophy experience both an increased bone breakdown and an impaired bone formation, presenting significant treatment challenges due to the dual pathology [6] (Table 1).

Muscle weakness, myopathy, and calcifications are additional components of CKD-MBD.

Bone strength depends on both its quantity and quality, which are influenced by various factors such as bone remodelling and matrix composition. The bone mineral density estimates the quantity, whereas microarchitecture assesses the quality, both of which contribute to a reduced bone mechanical strength in CKD. As a result, most fractures occur in bones with a low mechanical strength, often due to minor traumas like falls. Research has shown that individuals with Chronic Kidney Disease (CKD) face a heightened fracture risk due to a combination of factors, including CKD-related changes in bone and mineral metabolism, age, gender, diabetes, and glucocorticoid use [7]. In individuals over 65 years old with chronic kidney disease (CKD), women are more likely to experience fractures, with 1 in 10 women and 1 in 20 men suffering a fracture within three years [8]. The risk of hip fractures is four times higher for dialysis patients compared to the general population, even after adjustment for age, gender, and ethnicity [9,10]. CKD patients with severe kidney damage (G4 and G5) who experience hip and non-hip fractures have a higher mortality risk [11]. A large epidemiologic study in South Korea covering 352,624 CKD adult patients found lower eGFR and high levels of albumin, which was associated with an increased risk of hip fractures [12].

A review of existing research revealed 32 cases of isolated lesser trochanter (LT) fractures in adults, with 27 cases being pathological fractures. Notably, in 11 of these cases, the LT fracture was the initial sign of a previously undiagnosed malignancy [13]. LT avulsion fractures secondary to minor trauma in a patient with renal osteodystrophy have never been reported.

Avulsion fractures occur when a sudden contraction causes a piece of bone to break off at the point of a tendon or ligamentous attachment [14]. Common sites for this type of fracture include the greater and lesser trochanter of the femur, greater tuberosity of the humeral head, medial epicondyle of the elbow, ischial tuberosity, tibial tuberosity, and base of the fifth metatarsal. Sonography allows the visualization of avulsed bone fragments and their relationship to surrounding tissues. Typical signs of avulsion fractures on ultrasound include cortical discontinuity, local hematoma formation, and abnormalities in the surrounding soft tissues [15], which can vary depending on the location and severity of the injury.

Traditional imaging methods for bone evaluation include radiographs, CT, MRI, and scintigraphy (Table 2). Radiography remains the first-line investigation for diagnosing fractures, due to providing rapid identification of bony injuries. CT and MRI offer additional detail, particularly in complex or subtle cases.

HADD) in the tendons, which is also common in patients with renal osteodystrophy.

**Table 2:** Imaging features of Renal Osteodystrophy

Imaging Modality	Radiological Features of Renal Osteodystrophy
**Radiography**	- Osteosclerosis (especially in the axial skeleton) - ‘Rugger jersey spine’ (alternating bands of sclerosis and lucency in vertebrae) - Subperiosteal bone resorption, most notably in the hands (acro-osteolysis) - Brown tumours (osteolytic lesions) - Soft tissue and vascular calcifications
**CT**	- Diffuse osteosclerosis - Brown tumours appearing as lytic bone lesions - Cortical tunnelling and thinning - Subperiosteal and subchondral bone resorption
**MRI**	- Bone marrow oedema due to metabolic imbalances - Soft tissue involvement in brown tumours - Imaging of amyloid deposition (linked with long-term haemodialysis)
**Bone Scintigraphy**	- Increased uptake in areas of high bone turnover (e.g., brown tumours) - Distribution of metabolic bone disease across the skeleton, Super scan

*Ultrasound* (US) can serve as a complementary modality in assessing associated soft tissue injuries and detecting some occult fractures when radiographs are inconclusive [14,16–18]. However, its role is largely adjunctive, especially in cases where fractures are already evident on radiographs, as was in our case.

In bone imaging, ultrasound visualizes only the cortical surface as a bright (hyperechoic) line, without the ability to assess trabecular architecture [14,23,24]. While ultrasound can occasionally help identify periosteal changes, hematoma, or tendon involvement, definitive diagnosis of avulsion fractures typically relies on radiography, CT, and MRI. In our patient, radiographic and cross-sectional imaging findings were sufficient to confirm the diagnosis, with ultrasound providing supportive but non-essential information.

Uraemic tendinopathy is a condition observed in patients with chronic renal failure, particularly those undergoing long-term haemodialysis. It can lead to spontaneous tendon ruptures, such as quadriceps or Achilles tendon ruptures, due to the altered metabolic state associated with renal insufficiency. Although the exact pathophysiology remains unclear, factors like secondary hyperparathyroidism and uremia contribute to tendon weakening. The cases reported involved spontaneous ruptures, suggesting a possible link between chronic renal failure and tendon pathology, leading to the concept of ‘uremic tendinopathy’ [29].

In our case, the left triceps avulsion fracture was quite obvious, with the loss of elbow extension against gravity with a palpable gap in the tendon. Hence, the left elbow was immobilized in a plaster back slab, and only the right elbow ultrasound scan was performed at the time of presentation. Radiographs, CT, and MRI were helpful in confirming the diagnosis of avulsion fractures and excluding the differential diagnosis of *Hydroxyapatite Deposition Disease* (Take-away Points:

- Renal osteodystrophy in ESRD leads to weakened bones and increases the risk of atraumatic fractures, including rare avulsion injuries.
- Radiography and CT are primary modalities for diagnosing avulsion fractures; ultrasound plays a supportive role when needed for soft tissue assessment.
- Classical signs like brown tumours may be absent; diffuse sclerosis and subperiosteal resorption can still indicate renal osteodystrophy.
- Biochemical markers (e.g., PTH, calcium, phosphate, alkaline phosphatase) are critical in determining the type of renal osteodystrophy and guiding treatment.
- Treatment should be tailored to fracture severity, ranging from conservative management to surgical fixation for displaced injuries. The presented case underscores the importance of recognizing uremic bone disease in young ESRD patients with atraumatic fractures.
